# The combination of PD-L1 expression and the neutrophil-to-lymphocyte ratio as a prognostic factor of postoperative recurrence in non-small-cell lung cancer: a retrospective cohort study

**DOI:** 10.1186/s12885-023-11604-9

**Published:** 2023-11-14

**Authors:** Hironobu Samejima, Kensuke Kojima, Ayako Fujiwara, Toshiteru Tokunaga, Kyoichi Okishio, Hyungeun Yoon

**Affiliations:** 1 Department of General Thoracic Surgery, National Hospital Organization Kinki-Chuo Chest Medical Center, 1180 Nagasone-Cho, Kita-Ku, Sakai-Shi, Osaka, 591-8555 Japan; 2 Clinical Research Center, National Hospital Organization Kinki-Chuo Chest Medical Center, Osaka, Japan; 3 Department of Thoracic Oncology, National Hospital Organization Kinki-Chuo Chest Medical Center, Osaka, Japan

**Keywords:** Multivariable Cox proportional analysis, Neutrophil-to-lymphocyte ratio, Programmed death-ligand 1, Recurrence-free survival

## Abstract

**Background:**

While PD-L1 expression and neutrophil-to-lymphocyte ratio (NLR) are prognostic biomarkers for lung cancer, few studies have considered their interaction. We hypothesized that the product of PD-L1 expression (tumor proportion score) and the NLR (PD-L1 × NLR) might be a postoperative prognostic marker reflecting the immune microenvironment of lung cancer.

**Methods:**

We analyzed the association between PD-L1 × NLR and postoperative recurrence-free survival in 647 patients with NSCLC using multivariable Cox proportional hazards models.

**Results:**

In the analysis of PD-L1 × NLR as a categorical variable, the group with PD-L1 × NLR ≥ 25.8 had a significantly higher hazard ratio (HR) than the group with < 25.8 (adjusted HR 1.78, 95% confidence interval [CI] 1.23–2.60). The adjusted HR for PD-L1 × NLR, considered a continuous variable, was 1.004 (95% CI, 1.002–1.006). The risk of postoperative recurrence increased by 1.004-fold for each unit increase in PD-L1 × NLR, and a more than 2-fold increase in risk was observed for values ≥ 170.

**Conclusions:**

PD-L1 × NLR may be used in real-world clinical practice as a novel factor for predicting the risk of postoperative recurrence after lung cancer surgery.

**Supplementary Information:**

The online version contains supplementary material available at 10.1186/s12885-023-11604-9.

## Background

Primary lung cancer is the leading cause of cancer-related deaths worldwide [1]. Although complete surgical resection is recommended as a cure, a high rate of postoperative recurrence in advanced-stage lung cancer has also been reported [2]. Since the development of immunotherapy in recent years, the effects of immune checkpoint inhibitors on non-small-cell lung cancer (NSCLC) have attracted considerable attention [3]. In the surgical field, studies have shown that the use of immune checkpoint inhibitors in preoperative and postoperative adjuvant chemotherapy is associated with a favorable prognosis [4, 5]. Therefore, immunotherapy may play a central role in the treatment of NSCLC.

Programmed death-ligand 1 (PD-L1) is widely recognized as a biomarker for predicting the efficacy of PD-1 axis therapies [6]. PD-L1, which is expressed on a variety of malignant tumor cells, including NSCLC cells, is a ligand for programmed death 1 (PD-1), a molecule expressed on immune cells that negatively regulates the immune system [7]. PD-L1 expressed on tumor cells binds to PD-1 on immune cells and suppresses their activation. It is considered that expression of PD-L1 in tumor cells is considered to be associated with a poor prognosis, as such lesions tend to show malignant tumor growth via immunosuppression. Several studies have demonstrated that the expression of PD-L1 is a negative prognostic factor following lung cancer surgery [8–11], which corroborates this theory. This finding was further supported by our previous findings [12]. However, since there are lung cancer patients with high PD-L1 expression who have a good postoperative course in clinical practice [13], while other lung cancer patients with low PD-L1 expression may show a poor postoperative course in clinical practice [14], factors related to tumor immunity other than PD-L1 may also have a significant impact on postoperative prognosis.

Accumulating evidence indicates that a systemic inflammatory response is associated with poor prognosis in a variety of solid tumors, including NSCLC [15]. Neutrophils are attracted to the tumor stroma by chemokines, which are inflammation-related factors secreted by tumor cells. These attracted neutrophils are involved in the establishment of an inflammatory tumor microenvironment. Neutrophils are indirectly associated with angiogenesis, metastasis and inhibition of apoptosis through inflammatory responses in the tumor microenvironment, which consequently promote tumor growth [16]. Conversely, tumor-infiltrating lymphocytes exert anti-tumor effects and inhibit tumor growth [17]. Therefore, the neutrophil-to-lymphocyte ratio (NLR), which can be easily calculated by dividing the number of neutrophils by the number of lymphocytes, has attracted attention as a biomarker of tumor-related immune responses [18, 19]. Some studies have reported that NLR may be a predictive factor for lung cancer recurrence after surgery [20–22].

Both the expression of PD-L1 as a marker of tumor immunosuppression and NLR as a marker of the tumor immune response may be predictors of the risk of postoperative recurrence. In previous studies, the association between the expression, PD-L1 and the NLR with lung cancer prognosis was evaluated separately [13, 23–26]. However, no study has numerically addressed the interaction between PD-L1 expression and the NLR or evaluated the association between this interaction and the postoperative prognosis in NSCLC.

We hypothesized that the value of the product of the tumor proportion score (TPS), which represents the expression of PD-L1, and the preoperative NLR (subsequently “PD-L1 × NLR”) may be a new factor that better reflects the immune microenvironment of lung cancer, while more accurately predicting postoperative recurrence than either of these values alone. The association between this number and the postoperative prognosis of lung cancer was statistically evaluated. The value of PD-L1 × NLR, considering the immune microenvironment of lung cancer, may provide more accurate information for predicting postoperative recurrence in real-world clinical practice than either of these values alone.

## Methods

### Patients

A total of 647 patients with NSCLC who underwent complete surgical resection at Kinki-Chuo Chest Medical Center between January 2017 and April 2022 were included in our study. Complete resection was defined as a gross or microscopically removed tumor that corresponded to R0 in the residual tumor (R) classification. Patients with incomplete tumor removal (R1 resection) were excluded from the study. Our institutional pathologists performed histopathological diagnoses according to the 2015 World Health Organization classification. Platinum-based adjuvant chemotherapy was administered to eligible patients who provided informed consent based on the guidelines of the Japanese Lung Cancer Association.

Clinicopathological features, including age, sex, smoking status, comorbidities, histological type, pathological tumor-node-metastasis (TNM) classification (American Joint Committee eight edition), tumor size (invasive size), surgical procedure, adjuvant chemotherapy, and preoperative laboratory data (i.e., neutrophil counts and lymphocyte counts) were collected from the medical records.

The present study was approved by the Institutional Review Board of Kinki-Chuo Chest Medical Center (KCMC) (approval number: 2022–043). The Institutional Review Board of KCMC waived the requirement for informed consent from opting out of the research, which was provided on the homepage of KCMC. All methods were in accordance with the relevant guidelines and regulations.

### Tumor PD-L1 immunohistochemistry

All viable cancer cells were assessed in the entire pathological tissue section of each tissue sample. We evaluated PD-L1 expression using the PD-L1 clone 22C3 pharmDx kit and the Dako Automated Link 48 platform (Agilent Technologies, Dako, Carpinteria, CA, USA) and calculated the PD-L1 tumor proportion score (TPS) as the percentage of complete or partial membrane staining in a sample that included at least 100 viable tumor cells, ranging from 0 to 100%. The TPS calculation was performed following the standard 22C3 assay protocol. The tumor region was visually segmented into four areas, and the proportion of PD-L1-positive cells in each area was quantified, yielding an average value for the clinical TPS.

### Recurrence-free survival (RFS)

The primary outcome of this study was RFS, defined as the time from the date of curative surgical resection to the date of lung cancer recurrence diagnosis. The patients underwent blood examinations, chest radiography and chest computed tomography every three–six months. Further diagnostic procedures, such as magnetic resonance imaging (MRI) of the head, contrast computed tomography (CT), positron emission tomography (PET), and examination of tissue biopsy specimens, were performed when abnormal findings indicating possible disease recurrence were observed. The diagnosis of recurrence was based on a thorough clinical evaluation of these test results and was determined at a joint conference consisting of general thoracic surgeons, oncologists, radiologists, and pathologists.

### Statistical analyses

We used the Mann–Whitney U test to compare two unpaired groups with non-normal continuous variables. Pearson’s chi-squared test, which was used if the overall number of cases was ≥ 40, was used to compare categorical variables between non-recurrence and recurrence groups. Considering the product of PD-L1 expression (i.e., TPS) and the NLR, we were careful to treat TPS 0%. The value of PD-L1 × NLR will always be zero at 0% TPS and will not reflect the NLR at all. Therefore, TPS 0% was replaced by 0.5%, an intermediate value between 0%, and a detection limit of 1%. The predictive performance of the cutoff values of PD-L1 × NLR, PD-L1, and the NLR was calculated using the area under the receiver operating characteristic (ROC) curve (AUC). The probability of RFS was evaluated using the Kaplan–Meier method and the log-rank test.

We performed multivariable Cox proportional hazards analysis to estimate the hazard ratios (HRs) with adjustment for confounding factors. A multivariable Cox proportional hazards analysis can analyze the covariates of the number of cases with an outcome divided by 10 [27]. In the present study, the number of cases was 141 (number of cases with postoperative recurrence) divided by 10 (result:14). We included the value of PD-L1 × NLR as the explanatory variable of interest and the other 13 variables as confounding factors in the multivariable Cox proportional hazards model. Age, sex, histological type, pathological stage, tumor size, pathological N status, surgical procedure, and adjuvant chemotherapy were selected as confounding factors that have been reported to be potentially associated with postoperative recurrence of NSCLC [28–30]. In summary, we selected the following 14 factors as variables in a multivariable Cox hazards model: PD-L1 × NLR (≥ 25.8 [reference: < 25.8]), age (continuous variable), sex (female [reference: man]), histological type (squamous cell carcinoma (SCC) and other types [reference: adenocarcinoma (AD)]), pathological stage (stage II and III [reference: stage I]), tumor size (continuous variable), pathological N status (N1 and N2 [reference: N0]), surgical procedure (segmentectomy, lobectomy, and others [i.e., lobectomy with combined resection and pneumonectomy] [reference: wedge resection]), and adjuvant chemotherapy (platinum-based regimen [reference: none]).

We also evaluated the PD-L1 × NLR as a continuous variable rather than a categorical variable. In this case, the PD-L1 × NLR value as a categorical variable was replaced by a continuous variable. The proportional hazards assumption in the Cox models was qualitatively determined from Martingale residual plots. The presence or absence of multicollinearity in the variables of the multivariable Cox proportional hazards model was evaluated using a variance inflation factor (VIF) < 2.

All statistical analyses were conducted using Easy R (EZR) (Saitama Medical Center, Jichi Medical University, Saitama, Japan), a graphical user interface for R (The R Foundation for Statistical Computing, Vienna, Austria). EZR is a modified version of R commander with added biostatistical functions [31]. Statistical significance was set at *P* < 0.05.

## Results

### Patient characteristics

The clinicopathological characteristics of the 647 patients with completely resected NSCLC are summarized in Table 1. The median age of the patients was 71 years (range, 65–76 years). Men (58.8%) and smokers (64.4%) were significantly more common in the recurrence group than in the nonrecurrence group. Adenocarcinoma was the most common histological type (73.9%); however, there was a trend toward significantly more histological types other than adenocarcinoma in the recurrence group than in the non-recurrence group. Pathological stages I, II, and III were observed in 52 (36.9%), 40 (28.4%), and 49 (34.8%) patients in the recurrence group, and 404 (79.8%), 72 (14.2%), and 30 (5.9%) patients in the non-recurrence group, respectively. Patients in the recurrence group had a significantly higher number of advanced stages than those in the non-recurrence group. In the recurrence group with more advanced stages, extended resection (20 [14.2%]) was significantly more common than that in the non-recurrence group (16 [3.2%]). Adjuvant chemotherapy was administered to 65 (12.8%) and 37 (26.2%) patients in the non-recurrence and recurrence groups, respectively. Neutrophil counts (× 10^9^/L) were 3.0 (range, 2.4–3.9) and 3.5 (range, 2.8–4.4), and lymphocyte counts (× 10^9^/L) were 1.7 (range, 1.4–2.1) and 1.8 (range, 1.3–2.1) in the non-recurrence and recurrence groups, respectively. The expression of PD-L1, NLR, and PD-L1 × NLR were 2% (range, 0%–20%), 1.8 (range, 1.3–2.4) and 3.4 (range, 0.9–38.1) in the non-recurrence group and 15% (range, 1%–60%), 2.1 (range, 1.6–2.8) and 39.1 (range, 3.0–128.2) in the recurrence group, respectively.

**Table 1:** Baseline characteristics of patients

Characteristics	Overall (*n* = 647)	Non-recurrence (*n* = 506)	Recurrence (*n* = 141)	*P*
**Demographics**				
Age: median (range, Q1–Q3) (years)	71 (65–76)	71 (65–76)	71 (65–76)	0.87^c^
Male Sex: n (%)	381 (58.8)	280 (55.3)	101 (71.6)	< 0.001^d^
Current/Former Smoker: n (%)	417 (64.4)	310 (61.3)	107 (75.9)	0.001^d^
**Histological type: n (%)**				
AD	478 (73.9)	391 (77.3)	87 (61.7)	< 0.001^d^
SCC	116 (17.9)	85 (16.8)	31 (22.0)	
Others^a^	53 (8.2)	30 (5.9)	23 (16.3)	
**Pathological stage: n (%)**				
I	456 (70.5)	404 (79.8)	52 (36.9)	< 0.001^d^
II	112 (17.3)	72 (14.2)	40 (28.4)	
III	79 (12.2)	30 (5.9)	49 (34.8)	
**Pathological T status: n (%)**				
T1	434 (67.1)	372 (73.5)	62 (44.0)	< 0.001^d^
T2	151 (23.3)	98 (19.4)	53 (37.6)	
T3	47 (7.3)	29 (5.7)	18 (12.8)	
T4	15 (2.3)	7 (1.4)	8 (5.7)	
**Tumor size: median (range, Q1–Q3)**				
Invasive size (mm)	23 (15–32)	21 (14–29)	32 (23–43)	< 0.001^c^
**Pathological N status: n (%)**				
N0	538 (83.1)	458 (90.5)	80 (56.7)	< 0.001^d^
N1	56 (8.7)	30 (5.9)	26 (18.4)	
N2	53 (8.2)	18 (3.6)	35 (24.8)	
**Surgical procedure: n (%)**				
Wedge resection	31 (4.8)	25 (4.9)	6 (4.3)	< 0.001^d^
Segmentectomy	62 (9.6)	58 (11.5)	4 (2.8)	
Lobectomy	518 (80.1)	407 (80.4)	111 (78.7)	
Others ^b^	36 (5.5)	16 (3.2)	20 (14.2)	
**Adjuvant chemotherapy: n (%)**				
Platinum-based chemotherapy	102 (15.8)	65 (12.8)	37 (26.2)	< 0.001^d^
**Laboratory data before surgery: median (range, Q1–Q3)**				
Neutrophil count (× 10^9^/L)	3.1 (2.4–3.9)	3.0 (2.4–3.9)	3.5 (2.8–4.4)	< 0.001^c^
Lymphocyte count (× 10^9^/L)	1.7 (1.3–2.1)	1.7 (1.4–2.1)	1.8 (1.3–2.1)	0.71^c^
**Biomarker status: median (range, Q1–Q3)**				
PD-L1 expression: TPS (%)	5 (0–40)	2 (0–20)	15 (1–60)	< 0.001^c^
NLR	1.8 (1.4–2.4)	1.8 (1.3–2.4)	2.1 (1.6–2.8)	0.05^c^
PD-L1 × NLR	7.1 (1.1–61.3)	3.4 (0.9–38.1)	39.1 (3.0–128.2)	< 0.001^c^

*Abbreviations*: *AD* adenocarcinoma, *SCC* squamous cell carcinoma, *PD-L1* programmed death-ligand 1, *NLR* neutrophil-to-lymphocyte ratio, *PD-L1* × *NLR* the product of PD-L1 (TPS %) and the NLR

^a^Defined as histological types of NSCLC with the exclusion of AD and SCC. Among the 53 patients, 22 had pleomorphic carcinoma, 13 had large-cell neuroendocrine carcinoma, 11 had adenosquamous carcinoma, and 7 had large-cell carcinoma

^b^Defined as lobectomy with combined resection or pneumonectomy. Among the 36 patients, 31 underwent lobectomy with combined resection and five underwent pneumonectomy

^c^Mann–Whitney U test

^d^Pearson’s chi-square test

### The RFS

**Fig. 1:**
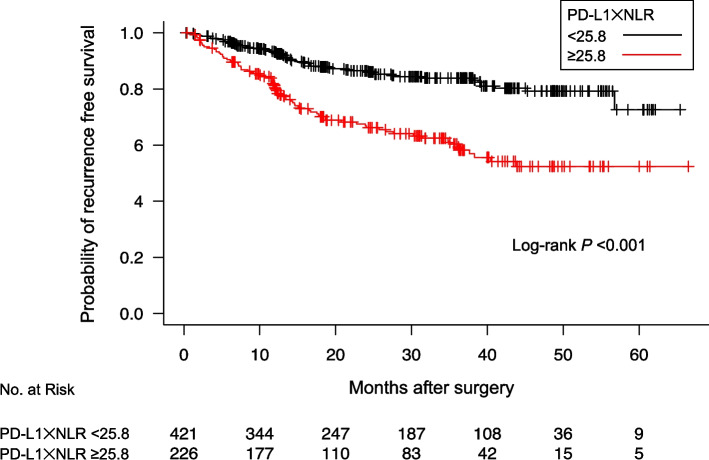
Kaplan–Meier curves showing the probability of recurrence-free survival among patients after surgery according to the value of PD-L1 × NLR. *NLR* neutrophil-to-lymphocyte ratio, *PD-L1* programmed cell death-ligand 1

During a median follow-up period of 729 days (360–1150 days) after surgery, 141 patients developed recurrent disease (21.7%). Receiver operating curve (ROC) analysis showed 25.8 a cutoff value of PD-L1 × NLR according to the RFS of patients with NSCLC (Supplemental Fig. S1a). In addition, ROC analyses showed 15.0 and 1.72 as the cutoff values of PD-L1 and the NLR, respectively, according to the RFS of patients with NSCLC (Supplemental Fig. S1b, c). The Kaplan–Meier curves for the RFS according to PD-L1 × NLR are shown in Fig. 1. The RFS in patients with a PD-L1 × NLR ≥ 25.8 was significantly shorter than in those with a value < 25.8 before adjusting for the patient background (log-rank *P* < 0.001). The Kaplan–Meier curves for the RFS according to the values of PD-L1 and the NLR are shown in Supplemental Fig. S2. The RFS in patients with PD-L1 ≥ 15.0 and NLR ≥ 1.72 were significantly shorter than in those with values < 15.0 and < 1.72, respectively, before adjusting for the patient background (log-rank P < 0.001).

### Multivariable analyses of postoperative RFS according to the PD-L1 × NLR

**Fig. 2:**
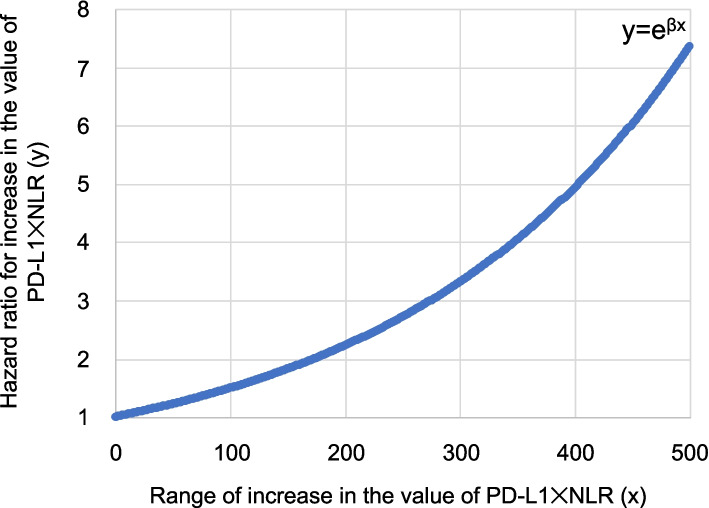
Hazard ratios of postoperative recurrence-free survival for each increase in the value of PD-L1 × NLR. The hazard ratio (y) for each value of PD-L1 × NLR to the range of increase from the value (x) was calculated based on the result of a multivariable Cox proportional hazards analysis. The hazard ratio for the regression coefficient (β) in this multivariable analysis was given by the value of e^β^, i.e., 1.004. The function y was given by y = e^βx^

The results of the multivariable Cox proportional hazards analyses for postoperative RFS according to PD-L1 × NLR as a categorical variable (cutoff value:25.8) are shown in Table 2. A significant difference in RFS was observed between the ≥ 25.8 group and < 25.8 group (adjusted HR, 1.84; 95% confidence interval [CI] 1.26–2.68) after adjusting for patient background factors. The proportional hazards assumption for this Cox model was assessed using a martingale residuals plot (Supplemental Fig. S3). The smoothed curve was generally horizontal, confirming that the proportional hazards assumption was satisfied. There was no multicollinearity in any of the explanatory variables used in the Cox model (VIF < 2) (Supplemental Table S1). Cox proportional hazards analysis, in which PD-L1 × NLR was considered a continuous variable rather than a categorical variable, showed that PD-L1 × NLR was significantly associated with postoperative RFS, with or without adjusting for patient background factors (unadjusted HR, 1.006; 95% CI, 1.004–1.007; adjusted HR, 1.004; 95% CI, 1.002–1.006) (Table 3). The proportional hazards assumption regarding this Cox model was also confirmed to be satisfied by the martingale residual plot (Supplemental Fig. S4). There was no multicollinearity in any of the variables in the Cox model (VIF < 2) (Supplemental Table S2). Based on the adjusted HR, the results of plotting the HR against each PD-L1 × NLR in the range of 0–500 are shown in Fig. 2. With the value of PD-L1 × NLR in the range of 0–100, the HRs ranged from 1–1.5. At ranges of 100–170, 170–340 and 340–500, the HRs ranged from 1.5–2.0, 2.0–4.0 and 4.0–7.5, respectively.

**Table 2:** Results of a Cox proportional hazard analysis of RFS according to the product of PD-L1 and the NLR as a categorical variable

	**Unadjusted HR (95% CI), *P***	**Adjusted HR**^**a**^** (95% CI), *P***
PD-L1 × NLR ^b^ < 25.8	Reference	Reference
PD-L1 × NLR ^b^ ≥ 25.8	2.77 (1.98–3.86), < 0.001	1.84 (1.26–2.68), 0.001

*Abbreviations*: *RFS* recurrence-free survival, *PD-L1* programmed death-ligand 1, *NLR* neutrophil-to-lymphocyte ratio, *HR* hazard ratio, *CI* confidence interval, *PD-L1* × *NLR* the product of PD-L1 (TPS %) and NLR

^a^Adjusted for age, sex, histological type, pathological stage, tumor size, pathological N status, surgical procedure, and adjuvant chemotherapy

^b^PD-L1 × NLR is a categorical variable

**Table 3:** Results of a Cox proportional hazard analysis of RFS according to the product of PD-L1 and the NLR as a continuous variable

	**Unadjusted HR (95% CI), *P***	**Adjusted HR**^**a**^** (95% CI), *P***
PD-L1 × NLR ^b^	1.006 (1.004–1.007), < 0.001	1.004 (1.002–1.006), < 0.001

*Abbreviations*: *RFS* recurrence-free survival, *PD-L1* programmed death-ligand 1, *NLR* neutrophil-to-lymphocyte ratio, *HR* hazard ratio, *CI* confidence interval, *PD-L1* × *NLR* the product of PD-L1 (TPS %) and NLR

^a^Adjusted for age, sex, histological type, pathological stage, tumor size, pathological N status, surgical procedure and adjuvant chemotherapy

^b^PD-L1 × NLR is a continuous variable

### Multivariable analyses of the postoperative RFS according to the PD-L1 and the NLR

As a control for the results of the multivariate Cox proportional hazards analyses of the postoperative RFS according to PD-L1 × NLR, we performed multivariable analyses of PD-L1 and the NLR alone. As PD-L1 × NLR, PD-L1, and the NLR differ greatly in the range of possible values, categorical variables rather than continuous variables were used for the comparison. The results of multivariable Cox proportional hazards analyses for the postoperative RFS according to PD-L1 and the NLR as a categorical variables (cutoff value: 15.0 and 1.72, respectively) are shown in Supplemental Tables 3 and 4. A significant difference in the RFS was observed between patients with PD-L1 values of ≥ 15.0 and < 15.0 (adjusted HR, 1.67; 95% CI, 1.15–2.43) and between those with an NLR of ≥ 1.72 and < 1.72 (adjusted HR, 1.59; 95% CI, 1.09–2.32).

## Discussion

In this study, we hypothesized that the product of the expression of PD-L1 (TPS [%]) in lung cancer and preoperative NLR (i.e., PD-L1 × NLR) is a novel prognostic factor that reflects the immune microenvironment in lung cancer. The association between the PD-L1 × NLR value and postoperative recurrence of NSCLC was analyzed using multivariable Cox proportional hazards models. The recurrence rate in our cohort was 21.7%, which is in close agreement with previous large cohorts reporting recurrence rates of 20–26% for lung cancer [32–34]. When PD-L1 × NLR was treated as a categorical variable, the cut-off value was 25.8. The group with PD-L1 × NLR ≥ 25.8 was associated with a significantly higher risk of postoperative recurrence than the group with PD-L1 × NLR < 25.8. When PD-L1 × NLR was treated as a continuous variable, the risk of postoperative recurrence was 1.004 times higher per unit of change. Based on this result, a mathematical model was developed to represent the risk of postoperative recurrence for each value of PD-L1 × NLR, and the results showed for the first time that the higher the value of PD-L1 × NLR, the greater the stepwise risk.

To assess the accuracy of the PD-L1 × NLR value as a risk factor for postoperative recurrence in direct comparison to individual single values, we conducted a comparison of Kaplan–Meier curves and HRs. The PD-L1, NLR, and PD-L1 × NLR variables differ greatly in the range of values that they can take. Therefore, we considered it unsuitable to use continuous variables for the comparison. To compare these three variables under identical conditions, we employed a binary variable, i.e. a categorical variable in this validation. Significant group differences were found for all variables in the Kaplan–Meier curves. Qualitatively, the difference between the PD-L1 × NLR and PD-L1 groups was greater than that in the NLR group. In the multivariable Cox proportional hazards analysis, the HRs for postoperative recurrence for the PD-L1 × NLR, PD-L1, and the NLR groups were 1.84 (95% CI, 1.26–2.68), 1.67 (95% CI, 1.15–2.43), and 1.59 (95% CI, 1.09–2.32), respectively. Quantitatively, the HR of the PD-L1 × NLR group was significantly the highest. These comparisons suggest that PD-L1 × NLR may offer greater utility as a biomarker for predicting the risk of postoperative recurrence than PD-L1 or the NLR individually.

The novelty of this study lies in the fact that both PD-L1 expression and the NLR were used as continuous variables and the clarification that their product value could predict postoperative prognosis in NSCLC. Previous studies on lung cancer prognosis have considered a combination of PD-L1 expression and the NLR [35–37]. However, PD-L1 expression and the NLR were treated as categorical variables and were evaluated in combination with each category in these reports. Therefore, the cut-off values differed among the studies, and the lack of uniformity in the combinations was considered problematic. We first performed a multivariable analysis to understand the association between the PD-L1 × NLR value and postoperative recurrence by setting cut-off values using ROC curves. The results showed that the group with PD-L1 × NLR ≥ 25.8 was at a higher risk than the group with PD-L1 × NLR < 25.8. However, this cut-off value was limited to the present study. Therefore, we evaluated the PD-L1 × NLR value using a continuous variable that was considered more reproducible. As a result, we have shown for the first time that higher PD-L1 × NLR values tend to increase the risk of postoperative recurrence. To our knowledge, this is the first study to show that PD-L1 × NLR is significantly associated with the risk of postoperative recurrence of NSCLC. This is a major new finding of this study, as it clearly demonstrates that a low NLR may reduce the risk, even with a high expression of PD-L1, whereas a high NLR may increase the risk even with a low expression of PD-L1.

There may be a biological explanation for the results of the present study. PD-L1 expression in tumors contributes to tumor promotion by suppressing the activity of tumor-infiltrating lymphocytes. Neutrophils have been reported to be associated with tumor promotion through their ability to assist tumor growth, angiogenesis and distant metastasis [38–40]. Furthermore, inflammation, reflected by an elevated NLR, also contributes to tumor promotion by stimulating the release of soluble signals associated with tumor growth, angiogenesis and metastasis [41]. Many tumor-infiltrating lymphocytes are thought to be cytotoxic T cells that play a central role in the antitumor immune response. It has been suggested that an increase in tumor-infiltrating lymphocytes, reflected by a decrease in the NLR, is associated with a favorable prognosis [42] and favorable effect of immune checkpoint inhibitors [43]. In addition, a decrease in the NLR may also reflect a decrease in inflammation. Inflammation is known to be associated with damage-associated molecular patterns (DAMPs), and it has been reported that DAMPs released by cancer cells induce myeloid-derived suppressor cells (MDSCs), which strongly suppress antitumor immune responses [44]. Thus, these findings suggested that high expression of PD-L1 and high NLR represent tumor-promoting properties, while low expression of PD-L1 and low NLR may conversely represent tumor-resistant properties. A low NLR may mitigate the tumor-promoting nature of the high expression of PD-L1 due to decreased inflammation and increased tumor-infiltrating lymphocytes. Conversely, a high NLR may counterbalance the weak tumor-promoting properties associated with low PD-L1 expression by increasing inflammation and decreasing the number of tumor-infiltrating lymphocytes. In our study, lymphocyte counts did not differ markedly between the nonrecurrence and recurrence groups. However, the NLR showed a significant difference between the non-recurrence and recurrence groups, indicating that the difference was highly dependent on the number of neutrophils. Changes in the NLR may better reflect the effects of inflammation than the properties of lymphocytes. It may be clinically relevant to consider the interaction between PD-L1 and the NLR, which are biomarkers from different compartments, from an immunological perspective. In essence, the value of PD-L1 × NLR may be a more accurate predictor of the risk of postoperative recurrence of lung cancer by better reflecting the immune microenvironment of the tumor than either of these values alone. PD-L1 is also expressed in immune cells [45]. In particular, PD-L1 expressed on tumor-associated macrophages (TAMs) and tumor-associated neutrophils (TANs) within the tumor microenvironment has been reported to contribute to the suppression of tumor-infiltrating lymphocytes [46, 47]. Interactions between TAMs and TANs have also been reported to contribute to tumor heterogeneity and proliferation [48]. Based on these findings, PD-L1 × NLR may also reflect interactions between neutrophils, lymphocytes, and macrophages within the tumor microenvironment that are relevant to tumor progression.

Several limitations of the present study warrant mention. First, this was a single-center observational study. A study with a larger cohort across multiple institutes is needed to assess the reproducibility of this study and to confirm the robustness of the results. Second, this was a single-center study with a small sample size. With regard to overall survival (OS), for which there were fewer outcome cases in comparison to RFS, OS was not examined in our study because sufficient removal of confounding factors could not be performed in the multivariable analysis. Further studies are needed to collect more cases and to determine whether there is a similar trend in OS. Third, theoretically, the PD-L1 × NLR value, which may reflect the immune microenvironment of the tumor, may be a predictor of the efficacy of immune checkpoint inhibitors. However, to confirm this assumption, the overall survival of immune checkpoint inhibitor-eligible patients needs to be evaluated, which is a future challenge. Fourth, there are limitations associated with the observational study design. Although we performed as much confounding adjustment as possible through multivariable analyses, the confounding introduced by bias due to differences in unmeasured factors between the groups remains an unaddressed issue.

## Conclusion

We used multivariable analyses to evaluate the association between postoperative recurrence of NSCLC and the PD-L1 × NLR, suggesting that the value of PD-L1 × NLR reflects the tumor immune microenvironment and may represent the risk of postoperative recurrence depending on its value. PD-L1 and the NLR are biomarkers that have already been used in real-world clinical practice, and it has been reported that each has the potential to become a factor that can independently predict postoperative recurrence of lung cancer. PD-L1 × NLR values may be applied as a predictor that can easily and accurately assess the risk of postoperative recurrence of NSCLC in comparison to each value alone.

### Supplementary Information

**Additional file 1:** **Supplemental Figure S1.** A receiver operating characteristic analysis to confirm the cut-off value ofPD-L1×neutrophil-to-lymphocyte ratio (NLR) (a), PD-L1 (b) and NLR (c) for the prediction ofpostoperative recurrence.

** Additional file 2:** **Supplemental Figure S2.** Kaplan–Meier curves showing the probability of recurrence-free survival among patients after surgery according to the value of PD-L1 (a) and NLR (b).

** Additional file 3:** **Supplemental Figure S3.** The evaluation of the proportional hazards assumption in Cox models.

** Additional file 4:** **Supplemental Figure S4.** The evaluation of the proportional hazards assumption in Cox models.

** Additional file 5:** **Supplemental Table S1.** Results of a Cox proportional hazard analysis of RFS according to the product of PD-L1 and the NLR as a categorical variable.

** Additional file 6:** **Supplemental Table S2.** Results of a Cox proportional hazard analysis of RFS according to the product of PD-L1 and the NLR as a continuous variable.

** Additional file 7:** **Supplemental Table S3.** Results of a Cox proportional hazard analysis of RFS according to the PD-L1 as a categorical variable.

** Additional file 8:** **Supplemental Table S4.** Results of a Cox proportional hazard analysis of RFS according to the NLR as a categorical variable.

## Data Availability

The datasets used and analyzed during the current study are available from the corresponding author upon reasonable request.

## Abbreviations

AD: Adenocarcinoma
HR: Hazard Ratio
NLR: Neutrophil-to-Lymphocyte Ratio
NSCLC: Non-Small Cell Lung Cancer
PD-L1: Programmed cell death-ligand 1
PD-L1 × NLR: Product of PD-L1 expression (tumor proportion score) and the NLR
RFS: Recurrence-free survival
SCC: Squamous cell carcinoma
TPS: Tumor proportion score
VIF: Variance inflation factor
95% CI: 95% Confidence interval
