# Supplementary material for: The combination of PD-L1 expression and the neutrophil-to-lymphocyte ratio as a prognostic factor of postoperative recurrence in non-small-cell lung cancer: a retrospective cohort study
Source: BMC Cancer. 2023 Nov 14;23:1107. doi: 10.1186/s12885-023-11604-9 (PMC10644552; doi:10.1186/s12885-023-11604-9)
Supplement: Supplementary file 1 — Additional file 1: Supplemental Figure S1. A receiver operating characteristic analysis to confirm the cut-off value ofPD-L1×neutrophil-to-lymphocyte ratio (NLR) (a), PD-L1 (b) and NLR (c) for the prediction ofpostoperative recurrence. [file 12885_2023_11604_MOESM1_ESM.pdf]

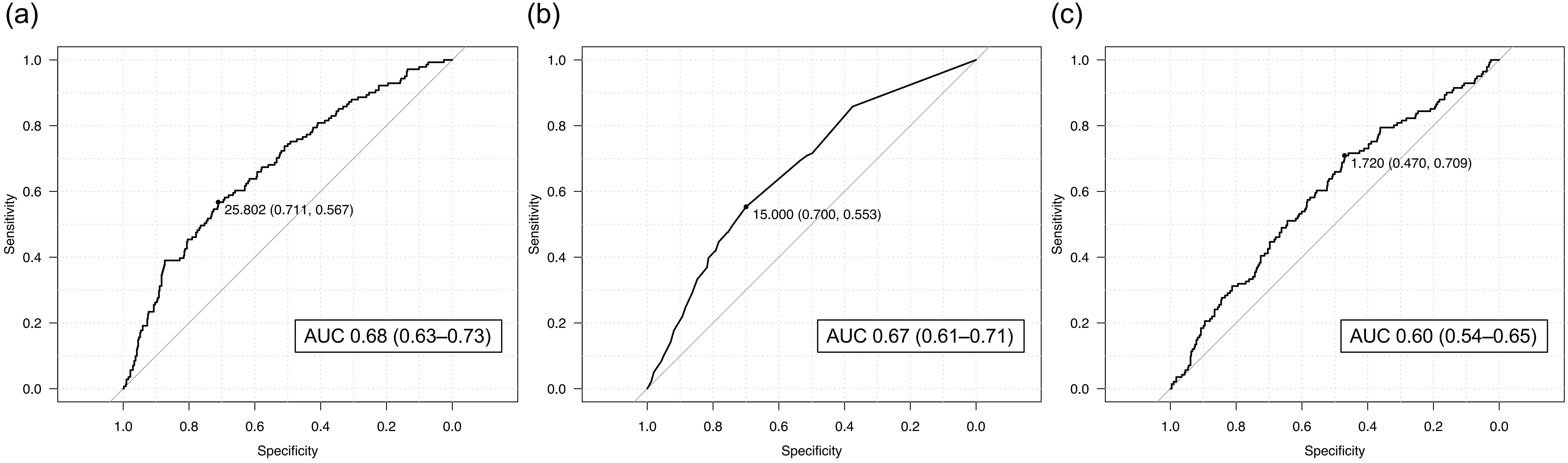

Supplemental Figure S1. A receiver operating characteristic analysis to confirm the cut-off value of PD-L1×neutrophil-to-lymphocyte ratio (NLR) (a), PD-L1 (b) and NLR (c) for the prediction of postoperative recurrence.

AUC area under the receiver operating characteristic
