# Supplementary material for: The combination of PD-L1 expression and the neutrophil-to-lymphocyte ratio as a prognostic factor of postoperative recurrence in non-small-cell lung cancer: a retrospective cohort study
Source: BMC Cancer. 2023 Nov 14;23:1107. doi: 10.1186/s12885-023-11604-9 (PMC10644552; doi:10.1186/s12885-023-11604-9)
Supplement: Supplementary file 2 — Additional file 2: Supplemental Figure S2. Kaplan–Meier curves showing the probability of recurrence-free survival among patients after surgery according to the value of PD-L1 (a) and NLR (b). [file 12885_2023_11604_MOESM2_ESM.pdf]

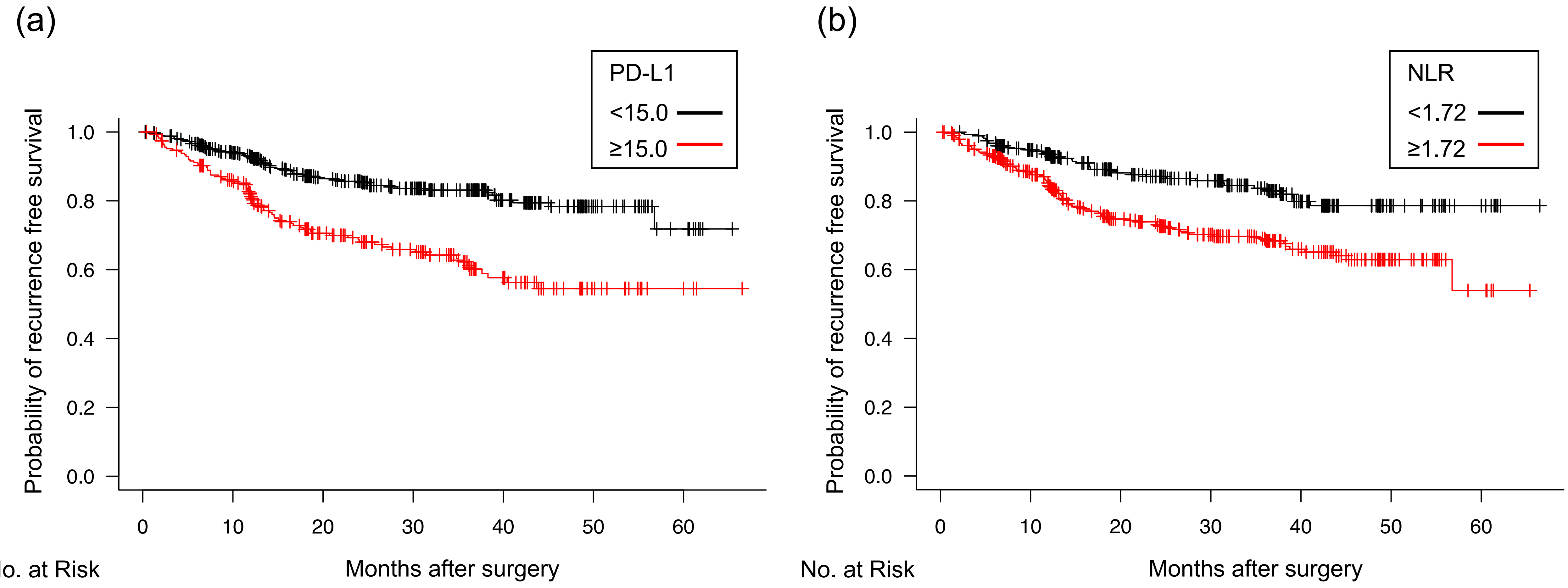

No. at Risk

PD-L1 < 15.0	417	339	240	183	105	34	9
PD-L1 ≥ 15.0	230	182	117	87	45	17	5

No. at Risk

NLR < 1.72	279	240	172	135	71	25	9
NLR ≥ 1.72	368	281	185	135	79	26	5

Supplemental Figure S2. Kaplan–Meier curves showing the probability of recurrence-free survival among patients after surgery according to the value of PD-L1 (a) and NLR (b).
AUC area under the receiver operating characteristic, NLR neutrophil-to-lymphocyte ratio, *PD-L1* programmed cell death-ligand 1
