# Supplementary material for: The combination of PD-L1 expression and the neutrophil-to-lymphocyte ratio as a prognostic factor of postoperative recurrence in non-small-cell lung cancer: a retrospective cohort study
Source: BMC Cancer. 2023 Nov 14;23:1107. doi: 10.1186/s12885-023-11604-9 (PMC10644552; doi:10.1186/s12885-023-11604-9)
Supplement: Supplementary file 4 — Additional file 4: Supplemental Figure S4. The evaluation of the proportional hazards assumption in Cox models. [file 12885_2023_11604_MOESM4_ESM.pdf]

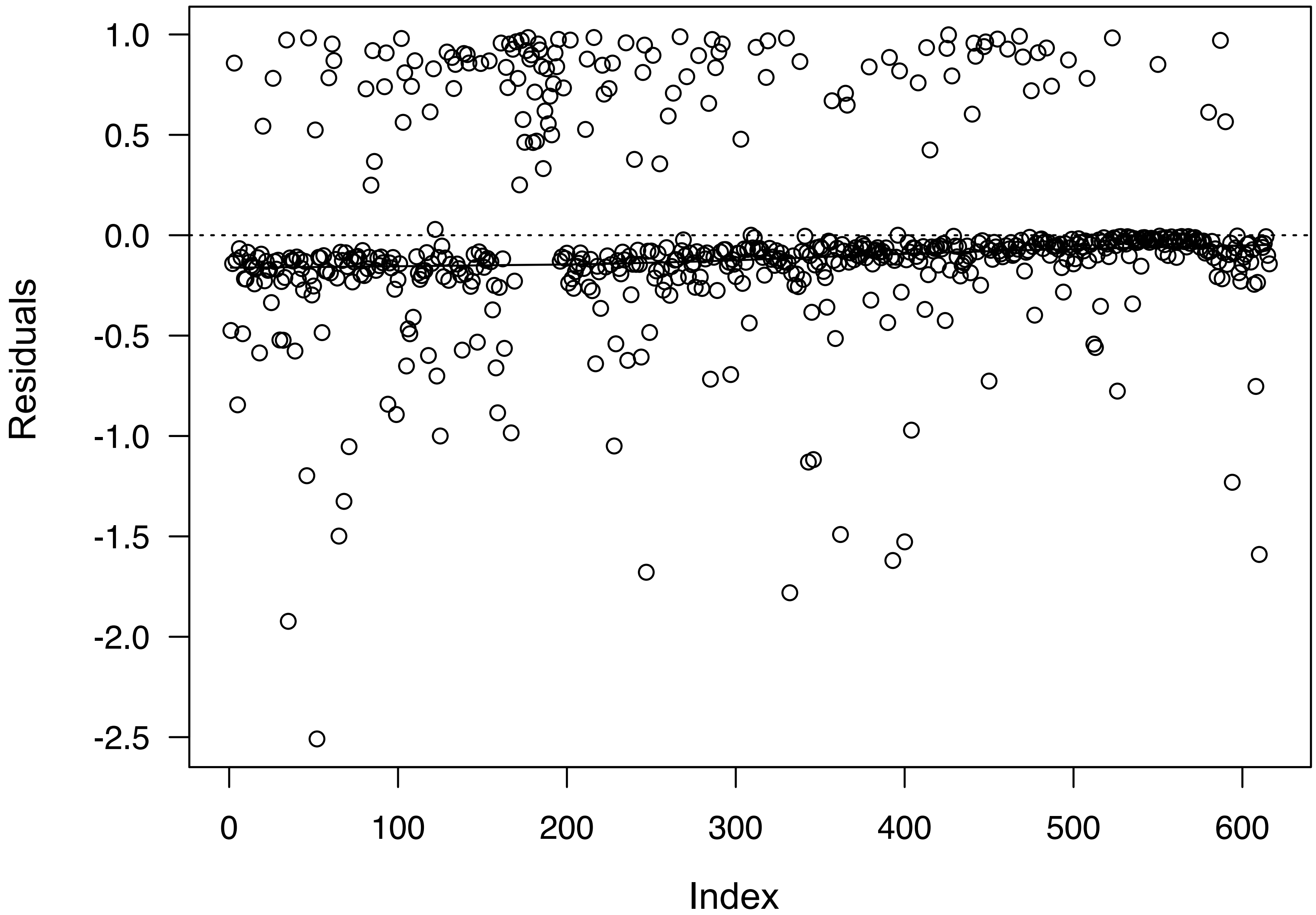


**Supplemental Figure S4. The evaluation of the proportional hazards assumption in Cox models.**

The proportional hazards assumption in Cox models was evaluated by a martingale residuals plot. The smoothing curve was generally horizontal.
