# Supplementary material for: The combination of PD-L1 expression and the neutrophil-to-lymphocyte ratio as a prognostic factor of postoperative recurrence in non-small-cell lung cancer: a retrospective cohort study
Source: BMC Cancer. 2023 Nov 14;23:1107. doi: 10.1186/s12885-023-11604-9 (PMC10644552; doi:10.1186/s12885-023-11604-9)
Supplement: Supplementary file 6 — Additional file 6: Supplemental Table S2. Results of a Cox proportional hazard analysis of RFS according to the product of PD-L1 and the NLR as a continuous variable. [file 12885_2023_11604_MOESM6_ESM.docx]

Supplemental Table S2. Results of a Cox proportional hazard analysis of RFS according to the product of PD-L1 and the NLR as a continuous variable.

|  | Unadjusted HR (95% CI), *P* | Adjusted HR ^a^ (95% CI), *P* | VIF |
| --- | --- | --- | --- |
| Continuous variable | | | |
| PD-L1×NLR ^b^ | 1.006 (1.004–1.007), <0.001 | 1.004 (1.002–1.006), <0.001 | 1.10 |
| Age | 1.003 (0.98–1.02), 0.78 | 1.01 (0.99–1.03), 0.23 | 1.08 |
| Tumor size | 1.021 (1.016–1.026), <0.001 | 1.013 (1.005–1.020), <0.001 | 1.59 |
| Categorical variables | | | |
| Sex  Men  Women | Reference  0.50 (0.35–0.72), <0.001 | Reference  0.68 (0.46–1.03), 0.06 | 1.07 |
| Histological type  AD  SCC  Others ^c^ | Reference  1.66 (1.10–2.50), 0.01  3.00 (1.89–4.76), <0.001 | Reference  1.01 (0.63–1.62), 0.97  1.65 (0.96–2.84), 0.07 | 1.11 |
| Pathological Stage  I  II  III | Reference  3.50 (2.31–5.28), <0.001  8.48 (5.73–12.56), <0.001 | Reference  1.42 (0.79–2.54), 0.23  2.54 (1.16–5.56), 0.02 | 1.78 |
| Pathological N status  N0  N1  N2 | Reference  3.65 (2.35–5.69), <0.001  7.06 (4.73–10.54), <0.001 | Reference  2.31 (1.26–4.24), 0.007  3.21 (1.49–6.89), 0.002 | 1.64 |
| Surgical procedure  Wedge resection  Segmentectomy  Lobectomy  Others ^d^ | Reference  0.32 (0.09–1.15), 0.08  0.92 (0.41­­­–2.10), 0.85  3.46 (1.38–8.63), 0.007 | Reference  0.21 (0.06–0.78), 0.02  0.52 (0.22­–1.23), 0.14  0.64 (0.23–1.75), 0.38 | 1.06 |
| Adjuvant chemotherapy  No adjuvant therapy  Platinum-based chemotherapy | Reference  1.77 (1.21–2.58), 0.002 | Reference  0.63 (0.39–1.02), 0.06 | 1.21 |

^a^ Adjusted for age, sex, histological type, pathological stage, tumor size, pathological N status, surgical procedure and adjuvant chemotherapy. ^b^ PD-L1×NLR is a continuous variable. ^c^ Defined as histological types of NSCLC with the exclusion of AD and SCC. Among the 53 patients, 22 had pleomorphic carcinoma, 13 had large-cell neuroendocrine carcinoma, 11 had adenosquamous carcinoma and 7 had large-cell carcinoma. ^d^ Defined as lobectomy with combined resection or pneumonectomy. Among the 36 cases, 31 underwent lobectomy with combined resection, and five underwent pneumonectomy.

*Abbreviations*: *RFS* recurrence-free survival*, PD-L1* programmed death-ligand 1, *NLR* neutrophil-to-lymphocyte ratio, *HR* hazard ratio, *CI* confidence interval, *VIF* variance inflation factor, *PD-L1×NLR* the product of PD-L1 (TPS %) and the NLR, *AD* adenocarcinoma, *SCC* squamous cell carcinoma
