# Supplementary material for: The combination of PD-L1 expression and the neutrophil-to-lymphocyte ratio as a prognostic factor of postoperative recurrence in non-small-cell lung cancer: a retrospective cohort study
Source: BMC Cancer. 2023 Nov 14;23:1107. doi: 10.1186/s12885-023-11604-9 (PMC10644552; doi:10.1186/s12885-023-11604-9)
Supplement: Supplementary file 7 — Additional file 7: Supplemental Table S3. Results of a Cox proportional hazard analysis of RFS according to the PD-L1 as a categorical variable. [file 12885_2023_11604_MOESM7_ESM.docx]

Supplemental Table S3. Results of a Cox proportional hazard analysis of RFS according to the PD-L1 as a categorical variable.

|  | Unadjusted HR (95% CI), *P* | Adjusted HR ^a^ (95% CI), *P* | | VIF |
| --- | --- | --- | --- | --- |
| Continuous variable | | | | |
| Age | 1.003 (0.98–1.02), 0.78 | 1.01 (0.99–1.03), 0.26 | | 1.09 |
| Tumor size | 1.021 (1.016–1.026), <0.001 | 1.013 (1.005–1.020), <0.001 | | 1.58 |
| Categorical variables | | | | |
| PD-L1 ^b^ <15.0  PD-L1 ^b^ ≥15.0 | Reference  2.47 (1.77–3.45), <0.001 | Reference  1.67 (1.15–2.43), 0.001 | 1.07 | |
| Sex  Men  Women | Reference  0.50 (0.35–0.72), <0.001 | Reference  0.64 (0.43–0.95), 0.03 | | 1.07 |
| Histological type  AD  SCC  Others ^c^ | Reference  1.66 (1.10–2.50), 0.01  3.00 (1.89–4.76), <0.001 | Reference  0.98 (0.61–1.59), 0.94  1.75 (1.03–2.99), 0.04 | | 1.11 |
| Pathological Stage  I  II  III | Reference  3.50 (2.31–5.28), <0.001  8.48 (5.73–12.56), <0.001 | Reference  1.52 (0.85–2.77), 0.23  2.60 (1.19–5.67), 0.02 | | 1.77 |
| Pathological N status  N0  N1  N2 | Reference  3.65 (2.35–5.69), <0.001  7.06 (4.73–10.54), <0.001 | Reference  2.25 (1.22–4.15), 0.009  3.07 (1.43–6.59), 0.004 | | 1.62 |
| Surgical procedure  Wedge resection  Segmentectomy  Lobectomy  Others ^d^ | Reference  0.32 (0.09–1.15), 0.08  0.92 (0.41­­­–2.10), 0.85  3.46 (1.38–8.63), 0.007 | Reference  0.27 (0.08–0.98), 0.05  0.49 (0.21­–1.16), 0.11  0.70 (0.26–1.93), 0.49 | | 1.06 |
| Adjuvant chemotherapy  No adjuvant therapy  Platinum-based chemotherapy | Reference  2.83 (1.91–4.18), <0.001 | Reference  0.65 (0.39–1.09), 0.11 | | 1.26 |

^a^ Adjusted for age, sex, histological type, pathological stage, tumor size, pathological N status, surgical procedure and adjuvant chemotherapy. ^b^ PD-L1 is a categorical variable. ^c^ Defined as histological types of NSCLC with the exclusion of AD and SCC. Among the 53 patients, 22 had pleomorphic carcinoma, 13 had large-cell neuroendocrine carcinoma, 11 had adenosquamous carcinoma and 7 had large-cell carcinoma. ^d^ Defined as lobectomy with combined resection or pneumonectomy. Among the 36 patients, 31 underwent lobectomy with combined resection, and five underwent pneumonectomy.

*Abbreviations*: *RFS* recurrence-free survival*, PD-L1* programmed death-ligand 1, *HR* hazard ratio, *CI* confidence interval, *VIF* variance inflation factor, and NLR, *AD* adenocarcinoma, *SCC* squamous cell carcinoma
