# Supplementary material for: The combination of PD-L1 expression and the neutrophil-to-lymphocyte ratio as a prognostic factor of postoperative recurrence in non-small-cell lung cancer: a retrospective cohort study
Source: BMC Cancer. 2023 Nov 14;23:1107. doi: 10.1186/s12885-023-11604-9 (PMC10644552; doi:10.1186/s12885-023-11604-9)
Supplement: Supplementary file 8 — Additional file 8: Supplemental Table S4. Results of a Cox proportional hazard analysis of RFS according to the NLR as a categorical variable. [file 12885_2023_11604_MOESM8_ESM.docx]

Supplemental Table S4. Results of a Cox proportional hazard analysis of RFS according to the NLR as a categorical variable.

|  | Unadjusted HR (95% CI), *P* | Adjusted HR ^a^ (95% CI), *P* | | VIF |
| --- | --- | --- | --- | --- |
| Continuous variable | | | | |
| Age | 1.003 (0.98–1.02), 0.78 | 1.01 (0.99–1.03), 0.35 | | 1.09 |
| Tumor size | 1.021 (1.016–1.026), <0.001 | 1.010 (1.003–1.018), 0.005 | | 1.58 |
| Categorical variables | | | | |
| NLR ^b^ <1.72  NLR ^b^ ≥1.72 | Reference  2.47 (1.77–3.45), <0.001 | Reference  1.59 (1.09–2.32), 0.02 | 1.03 | |
| Sex  Men  Women | Reference  0.50 (0.35–0.72), <0.001 | Reference  0.65 (0.44–0.96), 0.03 | | 1.07 |
| Histological type  AD  SCC  Others ^c^ | Reference  1.66 (1.10–2.50), 0.01  3.00 (1.89–4.76), <0.001 | Reference  1.15 (0.72–1.85), 0.54  2.01 (1.22–3.44), 0.006 | | 1.09 |
| Pathological Stage  I  II  III | Reference  3.50 (2.31–5.28), <0.001  8.48 (5.73–12.56), <0.001 | Reference  1.63 (0.91–2.91), 0.10  2.64 (1.21–5.78), 0.01 | | 1.77 |
| Pathological N status  N0  N1  N2 | Reference  3.65 (2.35–5.69), <0.001  7.06 (4.73–10.54), <0.001 | Reference  2.36 (1.26–4.29), 0.007  3.29 (1.54–7.07), 0.002 | | 1.62 |
| Surgical procedure  Wedge resection  Segmentectomy  Lobectomy  Others ^d^ | Reference  0.32 (0.09–1.15), 0.08  0.92 (0.41­­­–2.10), 0.85  3.46 (1.38–8.63), 0.007 | Reference  0.26 (0.07–0.93), 0.04  0.44 (0.19­–1.03), 0.06  0.62 (0.23–1.72), 0.36 | | 1.06 |
| Adjuvant chemotherapy  No adjuvant therapy  Platinum-based chemotherapy | Reference  2.83 (1.91–4.18), <0.001 | Reference  0.64 (0.38–1.08), 0.09 | | 1.25 |

^a^ Adjusted for age, sex, histological type, pathological stage, tumor size, pathological N status, surgical procedure and adjuvant chemotherapy. ^b^ PD-L1×NLR is a categorical variable. ^c^ Defined as histological types of NSCLC with the exclusion of AD and SCC. Among the 53 patients, 22 had pleomorphic carcinoma, 13 had large cell neuroendocrine carcinoma, 11 had adenosquamous carcinoma and 7 had large cell carcinoma. ^d^ Defined as lobectomy with combined resection or pneumonectomy. Among the 36 patients, 31 underwent lobectomy with combined resection, and five underwent pneumonectomy.

*Abbreviations*: *RFS* recurrence-free survival*, NLR* neutrophil-to-lymphocyte ratio, *HR* hazard ratio, *CI* confidence interval, *VIF* variance inflation factor, *AD* adenocarcinoma, *SCC* squamous cell carcinoma
